# Resilience and Wellbeing Strategies for Pandemic Fatigue in Times of Covid-19

**DOI:** 10.1007/s41042-022-00078-y

**Published:** 2022-09-30

**Authors:** Zachary Zarowsky, Tayyab Rashid

**Affiliations:** 1 Department of Psychology, University of Toronto Scarborough, 1265 Military Trial, Toronto, ON M1C 1A4 Canada; 2 Centre for Wellbeing Science, Melbourne Graduate School of Education, Melbourne, Australia

**Keywords:** Pandemic fatigue, Behavioural fatigue, Covid-19, Mental health, Wellbeing strategies, Resilience, Clinical psychology, Burnout

## Abstract

The COVID-19 pandemic is truly one of the greatest collective health crises in history which have altered our life and living. For years, people have felt fatigued from following public health directives such as social distancing, wearing masks, washing hands frequently, and working or studying remotely without in-person interactions. In this paper, we explore strategies for resilience and wellbeing which can mitigate pandemic-caused stress and behavioural fatigue. We start with individual level strategies including reworking stress appraisals, the importance of psychological flexibility, reducing loneliness through adaptive online platform use, optimizing familial relationships when living in close quarters for a prolonged period, reducing symptoms of burnout and using adaptive distractions, using specific evidence-based resilience strategies. We discuss specific considerations which tap on our shared identities and shared responsibilities which can enhance a sense of community, especially for individuals from marginalized backgrounds and how suicide risks can be minimized.

## Introduction

Rooted in biology, pandemics are complex events that produce a sequalae of social, economic, political, and most importantly, psychological impacts. The COVID-19 pandemic is truly an unprecedented and watershed event in generations which is also the greatest crisis of our lifetime. In March of 2020, the COVID-19 pandemic simultaneously forced nearly four billion people into mass quarantine, lock-down, or some form of home confinement (Sanford, 2020). Years of multiple lockdowns, stay-at-home orders, mask mandates, physical distancing, frequent hand washing, emerging variants, differential efficacy of vaccines, and significant lifestyle changes have disrupted almost all facets of life. A recent meta-analysis of 43 effect sizes from 36 studies found that globally, regardless of age, status as a medical provider, race, or region of origin, respondents experienced significantly elevated rates of psychopathology symptoms during the onset of the COVID-19 pandemic (Schafer et al., 2022). These rates escalated in the context of sharp economic challenges, long-term unemployment, closure of businesses, educational, cultural activities, and severe travel limitations, likely exacerbating overall stress.

While the adverse impact of the COVID-19 pandemic has been well documented (Armour et al., 2021; Osofsky et al., 2020; Pillay & Barnes, 2020), how individuals, communities, and countries have coped with it adaptively and effectively, has received far less scholarly attention. One notable exception being a set of recommendations related to pandemic fatigue by the World Health Organization (2020). Without discounting the distress caused by COVID-19, the goal of this paper is to share how wellbeing and resilience strategies can help individuals cope pandemics like COVID-19, given that the pandemic and its impact may linger for years. We begin strategies at the individual level such as psychological flexibility in changing the way we view stressors. We then discuss strategies to reduce loneliness and the influence of digital platforms. We follow this up with how to reduce relationships strain that comes from living in close proximity for extended periods of time together. We end the first section by exploring adaptive distractions and how we can enhance our resilience. Specifically, we highlight strategies which support the wellbeing of marginalized communities and mitigate risk of suicide, which historically has increased following epidemics and pandemics.

The ongoing COVID-19 pandemic with emerging variants has been inducing fear globally. Fear is among the most prevalent psychological responses to pandemics. A meta-analysis by Şimşir et al. (2021) of 33 different studies, from 20 countries, involving 70,407 participants showed that COVID-19 is significantly associated with mental health problems including depression, anxiety, distress, traumatization, and insomnia. Vaccines had buoyed the hope of people across the world (Mullard, 2021). However, the emergence of new and highly transmissible variants, which may not be treated fully with existing regiments of vaccines, have dashed the hopes and spirits of people as they feel tired, less motivated, and less efficacious — conditions which endanger behaviour fatigue (Haktanir et al., 2021). People feel exasperated at being asked to comply with directives which restrict their mobility and freedom, being strongly encouraged the avoidance of crowded places, required to remain masked and physically distanced while using public transport, and when using essential public services such as grocery shopping. Additionally, encouragement to stay and work from home while complying with wide-ranging restrictions for multiple years has severely exhausted many people’s ability to unbegrudgingly comply with health behaviour directives (Bell, 2020). However, Richard Jung and colleagues in their systematic review of 686 COVID-19 studies, compared with historical articles, found a shorter time to acceptance and lower methodological quality scores (Jung et al., 2021). This indicates that studies used to infer impacts on mental health should be interpreted within their given spatial and temporal context of COVID-19.

An illustration of these limitations is visible in emerging lines of research as demonstrated by a meta-analysis by Prati and Mancini (2021). They caution that the prevalent lack of control groups within many studies that have used cross-sectional designs and significant variation of socio-cultural contexts, present a significant methodological limitation on drawing conclusive inferences (Meda & Slongo, 2020). For example, it was found that the effect of lockdown on mental health symptoms were relatively small (g = 0.17, S.E. = 0.05, 95% CI (0.06–0.24). However, this could be a statistical artifact due to the small number of studies, significant heterogeneity of demographics, notable differences in length of lockdowns, and lack of sub-population analysis could mask the differential impact on sub-populations, such as children and frontline workers (Chen et al., 2020a, b; Prati & Mancini, 2021). Therefore, insights about the psychological impact of the pandemic from the emerging lines of research, at best, only offer broad and tentative conclusions. With these limitations in mind, we endeavor to explore the concept of behavioural and pandemic fatigue.

Behaviour fatigue can be described as the gradual decrease in desire to comply with a required behaviour over time due to factors such as boredom (Gever et al., 2021). Pandemic fatigue, a type of behaviour fatigue, is the tendency of feeling tired of following public health directives such as social distancing, wearing masks, washing hands frequently, and social isolation over an extended period of time (Bell, 2020; Thakre, 2021). Consistent with other behavioural fatigues, pandemic fatigue is characterized by reduced compliance with public health directives over time. Various conditions impact the experience of fatigue including age, gender, physical condition, type of food available, mental status, psychological conditions, personality and health status (Kim & Crimmins, 2020; Lai et al., 2020; Matias et al., 2020; Yao et al., 2020; Zhong et al., 2020). Nonetheless, required to follow public health directive—a top-down behaviour change approach would have repercussions and varied reactions. Not everyone is likely to adhere (Ngonghala et al., 2020). Furthermore, increasing psychological distress including depression would also create differential responses (Ettman et al., 2022).

Following specific public health directives, in particular social distancing and self-isolation is tough to follow over an extended period, especially when under increased psychological distress. This is because we are a deeply social species with both the quality and quantity of our daily face-to-face social interactions shaping our wellbeing (Sun et al., 2020). By interacting with others, we make sense of ourselves and the world around us. Our brains have evolved for socialization in a variety of diverse ways and situations, particularly when we are distressed or in pain (Eisenberg & Eggum, 2009). This in part explains why changing our behaviour has been particularly challenging, especially on a long-term basis.

With multiple lock downs, compulsory shelter-in-place orders, working from home, and the nation wide-spread closure of facilities — most psychological research on the COVID-19 pandemic has been on mental health outcomes. Reviewing the psychological impact of quarantine, Brooks et al. (2020) examined 24 studies and found that psychological impact of quarantine is wide-ranging, significant, and can be long lasting. After spending and adapting to varying degrees of social restrictions, many feel fatigued by being hunkered down in the confines of our homes. As we enter various stages of recovery and reopening, adapting to ongoing post pandemic realities has not been an easy or smooth transition which warrants further examination in the context of pandemic fatigue.

Bell (2020) highlights the following factors associated with behaviour-fatigue: people becoming irritated with regulations the longer they have to abide by, and eventually making a decision that they don’t want to comply with them; the degree to which people miss their friends and family, not being able to participate in social, cultural and leisurely activities for an extended period may encourage them to reduce compliance; people may become increasingly susceptible to those in their social circle who advocate a libertarian ideology that interprets government restriction as an infringement on their freedom that they don’t have to comply with; and people may erroneously assume that risk of infection has declined due to mass vaccination drives, and weaker variants – meaning they may start considering compliance and restrictions unnecessary. Additionally, as more and more people have been vaccinated and are hoping to resume affairs of life as we once knew, it is important to develop a deeper and more nuanced understanding of behavioural changes and their psychological impact. With these concerns in mind, we share the following insights derived from peer-reviewed research with an interpretive clinical lens.

## Resilience and Wellbeing Strategies for Pandemic-Fatigue

The multi-faceted causes of pandemic fatigue are often rooted in our innate needs and institutional frameworks not designed to accommodate these needs (Haktanir et al., 2021; Pandey et al., 2020). From cultural views that one’s health is a personal choice to the rejection of the idea that individuals bear little or no responsibility for the health of the community at large highlights that many countries were not prepared logistically or psychologically for the possibility of an extended pandemic of this nature. The combination of conflicting self-interests, fear, extended length of sacrificed time, underestimating the severity of COVID-19, isolation, misinformation, resistance to authority, societal inequity in resources, lack of governmental preparedness, and often seemingly contradictory information created ideal conditions for frustration and fatigue with public health restrictions to fester.

In the race between vaccines and variants, mass quarantine – especially at epicentres and hotspots, following rules and guidelines has been not easy. Compliance with authority is harder during a pandemic and could be especially irritating in societies where individual rights and freedoms are enshrined in the constitution and protected by law. Given that most people have had to deal with the psychological impact of pandemic related policies, it is imperative that skills and methods be available for individuals to deal with additional pressures.

Despite the finding by Prati and Mancini (2021) that the effect of lockdowns on mental health symptoms was small and significant, there was also large heterogeneity which could indicate that there are different psychological effects depending on social context, demographics, and region. This idea is potentially supported by the findings in both univariate and multivariate analysis that average age, length of lockdown, gender, study design, and publication status did not moderate the presence of psychopathology (Prati & Mancini, 2021).With that in mind, we discuss these areas in terms of strategies that can be implemented individually, including how one can appraise stress by incorporating flexibility, reducing loneliness using social media adaptively, enriching the quality of familial relationships, how one can mitigate burnout, suicidal behaviour, and the differential impact on marginalized communities. These areas were selected in light of emerging research and contextual relevance regarding the adverse effects of the pandemic on individuals (e.g., Haktanir et al., 2021). Additionally, although this article uses the most up to date information available, it should be noted that the combination of everchanging circumstances combined with the potential for variation by individual and contextual factors means that the presented strategies may not have equal relevance to all readers.

### Re-working Stress Appraisals Through Psychological Flexibility

One of the most common emotional responses during a pandemic is fear (Trnka & Lorencova, 2020). Humans respond to threats from their environment. Often these responses entail negative emotions such as fear. A meta-analysis found that targeting fear can be useful in some situations, but not in others. This is because appealing to fear leads people to change their behaviour if they feel capable of dealing with the threat but can also lead to defensive reactions when they feel helpless to act (Tannenbaum et al., 2015). Strong or intense fear produces the greatest behaviour change only when people feel a sense of efficacy and vice-versa, with stronger self-efficacy reducing fear (Hou et al., 2020a, b; Tannenbaum et al., 2015). As such, an important balancing act needs to be done internally regarding COVID-19 related fears to prevent people from either feeling that the risk to their own and loved one's health are overblown (too little fear) or that their COVID-19 related fears are so present and invasive that their ability to successfully perform in these burdensome circumstances are severely impaired (too much fear). An important concept that should be considered in this dynamic of how we respond to fear is psychological flexibility. Psychological flexibility is the tendency to change one's behaviour to be more functionally adaptive when placed in challenging situations (Doorley et al., 2020; Hayes et al., 2004, 2005).

#### Emerging Evidence

Given COVID-19 has adversely affected the psychological health of individuals by leading to stress, anxiety, panic disorders, and behavioral problems (Yildirim & Arslan, 2020), it is important to identify protective factors that reduce the negative mental health impact on individuals. Seiter and Curran (2021) have found that cognitively flexible individuals adapt more effectively to meet social needs in novel ways during the pandemic and consequently, adhere more to social distancing guidelines. This makes sense given the finding that those who are optimistic and psychologically flexible tend to be more resilient and have better coping resources to respond to adverse situations (Arslan et al., 2020). This finding has been reinforced by multiple studies as the COVID-19 pandemic has continued (Arslan & Allen, 2021; Casali et al., 2021; Liu & Wang, 2021; Umucu et al., 2021). Conversely, McCracken et al. (2021) have found that low psychological flexibility was associated with increased levels of depression, anxiety, and insomnia during the COVID-19 pandemic (Wąsowicz et al., 2021).

#### Practical Applications

In terms of approach coping strategies (e.g., positive reframing, active coping, acceptance, planning, etc.), the only strategies found to be associated with higher psychological flexibility were characterized by acceptance and positive framing (Rueda & Valls, 2020; Tindle et al., 2022). To implement these strategies, one can distancing themselves from potential negative outcomes related to COVID-19 in the future by grounding oneself in the present (White et al., 2019). Additionally, we can perceive potential negative outcomes through a logical lens instead of an emotional one (Robertson & Codd, 2019). These strategies allow us to view potential future pandemic events in a more constructive and pragmatic manner. For example, instead of focusing on the worst-case scenarios of what might happen if you or your loved ones contract COVID-19 and the fear that accompanies such thoughts, you can focus on exercising whatever measures and precautions you can to treat or prevent a worst-case scenario. To expand on this idea in a different context, people have found it useful to focus on the communal feeling of everyone working through the same pandemic restrictions as a way of reducing the perceived individual burden of limitations to collective aversity. Additionally, people have described being able to focus on higher quality interactions with those who they can socialize with as a way of coping (Sandbakken & Moss, 2021).

### Reducing Loneliness Through Virtual Connections

During crises, seeking social support is often one of the most adaptive ways to cope with stress (Elmer et al., 2020; McKinley, 2020; Sun et al., 2020). The COVID-19 pandemic has put many in a unique situation where this vital way of coping has been impeded, leading to social isolation and feelings of loneliness. To understand the spectrum of how pandemic-related lockdown, quarantine and social distancing measures impact mental health related to our ability to socialize, we must differentiate between social isolation and loneliness. Loneliness is the subjective and negative evaluation of the gap between one’s desired, and actual quality and quantity of social relationships (Peplau et al., 1979). Social isolation, on the other hand, is the actual number of social relationships one has in their social network (Weiss, 1973).

One potential strategy to partially reduce the impact of physical social isolation is the use of online connections that are informationally rich, dynamic, and temporally synchronous to attain social support. Although there has been contention that the overuse of the internet for socialization can increase social isolation due to online interactions lacking the qualities of in-person socialization (Costa et al., 2019), COVID-19 pandemic restrictions have rendered that particular downside a moot point in situations where in-person socialization is not feasible.

That being said, while Zoom and other video conferencing platforms enable us to have online communication, these platforms are not without limitations. For example, humans use a range of precisely timed vocalizations, gestures, and movements to communicate, and we rely on precise responses from others to determine if we are being understood. Though it appears that conversations are happening in real time, in reality there is slight (in milliseconds) delay between when an action is performed and when other participant receive it. Since people rely on providing and receiving specifically timed movements and vocalizations to indicate that they are being understood, more mental effort needs to be exerted to keep up with the delay in synchronous digital communication (Wiederhold, 2020).

#### Emerging Evidence

Making sense of an ongoing, unending, unpredictable pandemic, with ever changing rules and regulations is challenging. Public health guidelines strongly encourage people to distance socially, minimize social interaction or remain homebound, but for most people, these pandemic- related directives have resulted in new daily lives marked by decreased social interaction and increased stress, anxiety, and depressive states (Elmer et al., 2020; Ettman et al., 2022). As social isolation has varied throughout the pandemic, lockdown restrictions have in many cases also reduced the quality of supportive relationships leading to feelings of loneliness (Philpot et al., 2021).

Groarke et al. (2020) found that during the initial stages of the pandemic there were high rates of loneliness and that high levels of social support served as a protective factor.

 We should keep in mind that people may feel lonely, socially isolated, or both in specific domains of their lives. For example, not going to work, in person, to a stressful workplace, might be desired but not being able to see friends in person, could be stressful for the same person. Likewise, we ought to be mindful of individual differences. For example, some individuals with introvert personality attributes, those with signs and symptoms of social anxiety, and those with acute sensitivity to social comparisons and judgements. We need to avoid herd instinct and consider specific individualized social needs.

To stay in touch during the COVID-19 pandemic, people have flocked to various social media platforms (Ngien & Jiang, 2021). There have been mixed findings on the impact this increased use has had on mental health (Hou et al., 2020a, b), with some studies finding negative outcomes (Gao et al., 2020) and others suggesting potential for positive outcomes (Yousri et al., 2021). Positive outcomes primarily entailed staying connected with friends, peers and colleagues through platforms such as Zoom, Webex or WhatsApp. Additionally, for some, not having to deal with the potential social anxiety of in-person socialization was beneficial when being able to use more streamlined online interactions (Ho & Moscovitch, 2021).

In the context of youth, the increased use of internet throughout the pandemic to compensate for isolation from peers and extracurricular activities may have reduced time spent doing otherwise beneficial activities, potentially negatively impacting their psychological and emotional health (McDool et al., 2020; Panchal et al., 2021). This effect could have also been compounded by problematic internet usage as characterized by the overuse of social media, one’s smartphone, and excessive gaming (Chen et al., 2020a, b). These lines of research indicate that how one approaches social media use may influence mental health outcomes and, by extension, pandemic fatigue. Indeed, current circumstances bolster the suggestion by Nowland et al. (2018) that healthy internet use can have a positive impact on feelings of loneliness when used to form new relationships or improve existing ones instead of avoiding socialization.

Shao et al. (2021) suggest that using social media to disclose pandemic-related anxiety and fear does not relieve stress and leads to emotional burnout, possibly due to shared fears exacerbating pre-existing anxiety and emotional contagions. Conversely, people who are more likely to share positive information about COVID-19 tend to have higher life satisfaction and sense of adequacy (Yang et al., 2020). Shao et al. (2021) also found that individuals who are more skilled at regulating their emotions and positive attributes such as self-compassion, mindfulness, and cognitive reappraisal tend to seek supportive engagement more often with their networks. This difference in skills versus support resources is important because it could result in those with otherwise good emotional regulation skills having worse mental health than those of a similar skill level who are more easily able to contact their support network.

#### Practical Applications

Determining safe ways to overcome changes in our routines and fulfilling social support needs is critical for preventing feelings of loneliness. To that end, we can harness social media for positive coping. People should be informed of effective ways to improve emotional regulation and try to consume more emotionally positive or neutral content (Shao et al., 2021). For example, individuals can be advised to share digital literature (e.g., e-books) and content with people they know to make them happy, providing both a reason and topic to speak on (Rashid & McGrath, 2020). To manage or minimize problematic internet usage, Panchal et al. (2021) found that youth can be guided towards other activities such as reading and playing board games (Li et al., 2016), physical activities (Liu et al., 2021), and listening to music (Goldbeck & Ellerkamp, 2012). To avoid negative outcomes of video communication, such as Zoom fatigue, Baker and Murphy (2021) suggest looking at the camera, instead of other participants to establish direct eye contact and make the interaction feel more authentic.

Furthermore, during a crisis such as the COVID-19 pandemic, interventions, otherwise delivered in person, can be accessed virtually. A systematic review by Williams et al. (2021) on interventions designed to reduce social isolation and loneliness during the COVID-19 pandemic found that many potentially low-cost and digitally deliverable interventions are effective, including; mindfulness-based therapies, visual art discussions, laughter therapy, and Tai Chi Qigong meditation. It was also found that although there was much variation in the included educational programme interventions, multiple studies demonstrated the positive effects of addressing social integration barriers and making friends had on loneliness which can be targeted directly in a therapeutic context (Hawkley & Cacioppo, 2010; Williams et al., 2021). In terms of facilitating communication with others, the effectiveness of social facilitation interventions was found to be the most supported by evidence (Williams et al., 2021). This suggests that finding ways to improve relationships with existing contacts over making new friends may be more beneficial for those experiencing loneliness, but further research is needed to test reliability and validity of this claim and the effectiveness of said interventions. Additionally, any approach to address these issues must consider that those most likely to be experiencing social isolation or loneliness also potentially lack access or knowledge on how to use the technology needed to implement intervention delivery (Beaunoyer et al., 2020; Torous et al., 2020; Williams et al., 2021).

### Managing Relational Burnout

Lockdowns in Spring 2020 resulted in sudden and drastic changes in family living arrangements, including in some cases the loss of the ability to leave a hostile living environment and anticipatory grieving. Anticipatory grief refers to mourning the potential loss of a loved one before they die. The public safety measures associated with COVID-19 containment created additional complexity surrounding the process of death, dying, and mourning. It has also been found that Post-traumatic Stress Disorder (PTSD) among Intensive Care Unit (ICU) survivors is common and long-lasting, with previous studies having demonstrated a link between hospitalization during a pandemic with PTSD (Luyt et al., 2012; Wu et al., 2005). For example, in a study of long-term outcomes following the H1N1 pandemic in 2009, researchers found that at one year follow-up, between 41–44% of discharged ICU patients were at risk for PTSD (Luyt et al., 2012). This highlights that even though one may survive; the sheer length and slow speed of the recovery period can add to pandemic fatigue. More specifically, family members may experience anticipatory grief and components of ambiguous loss; both are factors known to be associated with worse outcomes, which in the context of the pandemic may add to pandemic-fatigue (Singg, 2009).

#### Emerging Evidence

Families have faced enormous challenges in their relationships during COVID-19 with upwards of one-third of families having reported feeling very or extremely anxious about stress resulting from COVID-19-related confinement (Statistics Canada, 2020). For example, for American students forced to either return home from or begin their post-secondary education online – Hall and Zygmunt (2021) found that although some were excited to return home and were welcomed by their parents, students commonly felt less adultlike and that there was a loss of autonomy. Additionally, a meta-analysis by Panchal et al. (2021) found that the risk factors for poor mental health outcomes in youth populations included how time on the internet was spent (Chen et al., 2020a, b), having a relative that works COVID-19-related front-line occupations, exposure to COVID-19 media (Chen et al., 2020a, b), and a lack of routine (Ren et al., 2021). As such, if not overburdensome, attending school can be a protective factor for youth by providing important daily structure to their lives (Wang et al., 2020). However, cross-sectional designs in addition to a lack of consistent symptom assessment and lockdown duration information hampered a sophisticated understanding of the differential impact of COVID-19 (Panchal et al., 2021). That being said, within the family paradigm, recent research by Swit and Breen (2022) found that corporal punishment, parental ability to negotiate parenting identity and role, unemployment and/or major loss of finances, and living in disadvantaged neighbourhoods have been psychological risk factors exacerbated by the realities of COVID-19.

Negative perceptions of one’s identity as a parent resulting from the above risk factors may distort parents' reaction to stressors (Epifanio et al., 2015), leading to parental burnout and potential maladaptive coping. Finding ways to counter parental burnout and stress during COVID-19 is important as they are a risk factor for child abuse and authoritative parenting behaviors that can increase the risk of parental violence (Anthony et al., 2005; Curtis et al., 2000; Swit & Breen, 2022). To that end, Swit and Breen (2022) found that at the familial level, parental emotional regulation and child independence were the strongest protective factors that alleviate parenting stress. This suggests that psychological interventions for families can focus on improving parents’ ability to regulate their emotions (Coyne et al., 2021), and to increase children’s independence from a young age.

#### Practical Applications

As public health directives have increased the amount of time spent with members of the same household, tension can build over time. To reduce this potential tension, it is recommended that relationships in the family be forged and maintained during the extra time spent together to offset the consequences of otherwise distressing situations such as loneliness (Prime et al., 2020). Along this line of thought, Prime et al. (2020) also recommend that collaboration within the family take place to reach a shared framework of how COVID-19 related stressors impact each family member differently to deepen compassion and understanding between members. In line with the finding that parental emotional regulation reappraisal is a strong predictor in protection from parental burnout during COVID-19, Swit and Breen (2022) suggest that parents can be explicitly advised to implement well-being strategies such as changing feelings and emotions through changing the way one appraises situations (Gross & John, 2003). A wellbeing and positive emotion-based perspective has the potential to improve the day-to-day experiences of parenting in trying times. Likewise, since children that are more dependent on their parents (such as infants) require more attention compared to older children that exercise greater autonomy and self-care, it is not surprising that the level of independence a child has was a strong predictor of parental burnout (Lindhal-Norberg, 2007; Lindström et al., 2011; Mikolajczak et al., 2018; Swit & Breen, 2022). Having older children in the context of COVID-19 has been generally advantageous for parents that have already been struggling to balance increased responsibilities as they are less likely to need to make meals for or entertain their children, and thus have more opportunities to take breaks (Mak et al., 2020; Swit & Breen, 2022). To that end, interventions to promote early childhood independence may provide an avenue to protect parents from parental burnout, in turn protecting the children from the negative consequences of parental burnout—a win–win scenario.

### Mitigating Burnout and Using Adaptive Distractions

Research has indicated that COVID-19 pandemic has amplified stress of a significant proportion of population (Panchal et al., 2021; Prati & Mancini, 2021). Excessive stress can notably lead to burnout, a prominent factor in pandemic fatigue (Marčinko et al., 2020; Taylor et al., 2022; Yildirim & Solmaz, 2020). Burnout is a psychological construct defined by the experience of emotional exhaustion, reduced personal accomplishment/inefficacy, and depersonalization/cynicism (Maslach & Jackson, 1981; Maslach & Leiter, 2016). Broadly speaking, individuals' conception of themselves and others influence how one experiences stressors (Maslach & Leiter, 2016), meaning that the idiosyncratic way that different people experience stress should be considered when trying to prevent burnout (Yildirim & Solmaz, 2020). This is reflected in the broad range of ways that burnout can negatively impact mental health, including; sleep issues, substance abuse, memory impairment, neck pain, anxiety, depression (Peterson et al., 2008), reduced productivity and motivation (Dugani et al., 2018), and reduced job satisfaction (Maslach et al., 2001).

Teaching people healthy coping and strategies that enhance resilience can help in mitigating the impact of negative symptomatic stress (Park et al., 2021a, b). Studies have shown that resilience (to be discussed in the next section) serves as a mediator between stress and burnout, meaning that resilience can mitigate the detrimental effect of stress on burnout (Deldar et al., 2018; Hao et al., 2015). Adaptive distraction and seeking emotional support have also been shown as effective coping strategies in young adults such as students when they have limitations on their behaviour such as physical distancing or staying home (Janson & Rohleder, 2017; Park et al., 2021a, b).

#### Emerging Evidence

With the increased presence of COVID-19, in many cases the workload of daily life has forced us to take on additional responsibilities (e.g., working from home while caring for children, healthcare workers, etc.). As discussed earlier, strategies which enhance psychological flexibility can mitigate the distress of COVID-19. This is especially true when one is able cultivate creative hopefulness, shift and contextualize, and focus on process instead on outcome and align tasks with values. Then accepting and tolerating negative feelings related with COVID-19 challenges becomes easier. Such strategies reinforce values-based behaviour despite adverse circumstances (Tindle et al., 2022). Practicing these strategies leave little room for avoidant coping (e.g., venting, self-blame, denial, etc.). In addition to psychological flexibility, limiting extended work hours, improving sleep quality, tapping onto one’s social supports, and optimism have been found to improve self-efficacy during the COVID-19 pandemic, which in turn reduces burnout (Hou et al., 2020a, b; Leśniewska et al., 2021).

#### Practical Applications

To put previous and emerging findings on mitigating burnout into practice, it is important to take regular breaks for oneself throughout the day, something that was embedded in traditional working environments and easily overlooked when working from home. Reflection through journaling and mediation can also be powerful tools to address burnout by making it easier to identify stressors that can then addressed (Jackson Preston, 2022). One can also reflect on an activity one finds meaningful and that can realistically be done under the current circumstances. Then one can formulate a clear plan regarding how they would start doing the activity the next day. Consider the necessary steps to ensure that they are ready to start pursuing the activity and to think about any obstacles that could stop them from doing this activity and how to overcome them, etc. (Oettingen et al., 2015). Psychoeducation can be used to identify maladaptive coping strategies such as substance abuse, excessive media consumption, brooding, procrastination and provide healthier alternatives (Hansel et al., 2020). In addition to the already discussed strategies, distraction can also be helpful as a coping strategy, especially in situations of low control (Park et al., 2020). Individuals can distract themselves by focusing on things which they can control.

Distraction can be both an adaptive and maladaptive coping strategy depending on how it is used. The key difference between the two is that distraction becomes adaptive when used in combination with an acceptance approach to stressors, whereas it is maladaptive when used to avoid cognitively confronting stressors at all (Wolgast & Lundh, 2017). What makes this difference adaptive is that the distraction draws an individual’s attention away from negative moods to allow for a safer, more neutral one, that allows for less painful processing of emotions (Van Dillen & Koole, 2007). In the context of COVID-19, adaptive distraction would entail intentionally or unintentionally drawing one’s attention away from the focal event (e.g., COVID-19 related stressors such as fear of infection, social distancing, quarantine).

Integrating these strategies into daily life can be challenging, particularly in environments that limit access to personal free time and resources. To that end, one may find it useful to develop an individualized wellness plan that integrates various strategies such as the ones suggested to reduce burnout (Jackson Preston, 2022; Kent, 2021; Mills et al., 2020; Parsons et al., 2020). Various free and easy to access tools exist that are designed to help people understand and develop their own personal wellness plan, such as the Professional Quality of Life (proQOL) Measure (Stamm, 2005, 2010) and University of Buffalo self-care starter kit (Butler & McClain-Meeder, 2015). With the assistant of these tools, individuals can easily assess the nature of their coping strategies (i.e., positive vs negative), determine which areas of personal wellbeing should be a priority (e.g., emotional, physical, social, spiritual), and explore effective ways to deal with potential barriers (e.g., lack of resources, number of commitments, social support) (Jackson Preston, 2022).

### Enhancing Resilience

While most of us have complied with pandemic directives, an intriguing question in the context of pandemic fatigue is how resilient are we in the face of an unpreceded and unabating pandemic? Resilience at its most basic conceptual level can be understood as the capacity for adapting to adversity or overcoming challenges (Masten, 2018; Smith et al., 2008). However, turning the abstract concept of reliance into practice is complex as it entails dynamic interactions involving numerous processes across developmental, social, cultural, economic and political systems (Masten, 2018). An example of what this might look like is the difference in how well a young adult versus an elderly person may bounce back mentally from infection with COVID-19, with the young adult more likely to recover quickly or use different coping strategies (Rashid et al., 2021). As the pandemic has gone on, it is more and more likely that most people will have had a firsthand encounter with personal infection or infection of loved ones. When this occurs, people may feel helpless and can easily resort to unhealthy coping strategies – particularly if they are unable to manage the care of themselves or loved ones (Jafari-Oori et al., 2021; Renzi et al., 2020).

#### Emerging Evidence

Reviewing emerging literature on the impact of the pandemic on mental health both in the short and long term, Manchia et al. (2022) have noted that by following public health directives, the pandemic has asked a lot from all of us. Their comprehensive review found increased levels of anxiety, depression, and a host of psychological problems have been reported (Armour et al., 2021; de Sousa et al., 2021). Nonetheless, a not insignificant portion of the population remained for the most part unaffected or were even flourishing under the new circumstances. Although longitudinal studies demonstrated encouraging signs of resilience, members of certain populations such as healthcare workers, students, children, and adolescents were found to be either more vulnerable to, or experiencing, increased mental health concerns (Manchia et al., 2022). However, Panchal et al. (2021) have also found that most children have been able to adapt to and cope with lockdown measures (92.6%; Pisano et al., 2020), with many family relationships improving throughout the lockdowns (41.6%; Graell et al., 2020) and children being happy to spend more time with family (58%; Shah et al., 2021). Healthy relations such as these were found to be associated with positive parent–child communication (Tang et al., 2021). Additionally, despite those with existing mental health disorders having experienced negative impacts on their mental health throughout the pandemic, most do not appear to have increased symptom severity compared to before the pandemic (Manchia et al., 2022). Furthermore, infection of COVID-19 has left many more vulnerable to developing a mental health condition (Thye et al., 2022).

#### Practical Applications

Practicing and enhancing resilience can be another strategy to deal with pandemic fatigue, as emerging evidence has shown (DeTore et al., 2021; Kelly et al., 2021; Park et al., 2021a, b). The findings presented by Manchia et al. (2022) highlight the need to use a personalized approach when identifying those within their unique context, who are more or less able to develop and exercise resilience in the face of COVID-19.

To resist the negative psychological impact of the COVID-19 pandemic, such as being in an Intensive Care Unit (ICU) for COVID-19 treatment (Shaygan et al., 2021), resilience interventions focus on helping people identify how their stressful experiences can be used to increase psychological toughness. This can take the form of helping people to reorganize priorities, identify what they can do and cannot do, and generally harness the stress response to focus on positive gains (Crum et al., 2013; Crum & Lyddy, 2014). Under these circumstances, viewing one’s stressors through a lens of mindfulness can allow that stress to be enhancing instead of debilitating (Crum et al., 2013; Crum & Lyddy, 2014). In the case of COVID-related stressors, this may mean acknowledging that the COVID-19 pandemic causes concerns and recognizing that it is within range of normal reactions to feel elevated stress related to the worry for loved ones’ health during a pandemic. By identifying what we can do instead of focusing on what we cannot do, we remain more grounded, which reduces feelings of being overwhelmed and helpless.

Resilience enhancing strategies during the COVID-19 pandemic can also vary based on unique stressors faced by different members of the community. For example, in families with children, parents can facilitate increased resilience in their children by practicing a structured and warm caregiving approach (Stark et al., 2020) along with spending even short periods of quality time with one's children (Bartlett & Vivrette, 2020). For healthcare workers, it has been found that practicing self-care by balancing work and personal life enhances one’s resilience. The role of cultivating and maintaining meaningful relationships is critical in this (Callahan et al., 2018; Heath et al., 2020). For example, Mai et al. (2021) found that high levels of perceived emotional support from friends and family directly translated to how well students were able to cope, indicating that students should aim to find sufficient social support from loved ones to serve as a facilitator to developing resilience. A six-week online resilience intervention which included expressive writing significantly enhanced resilience among adults who self-identified as having been significantly affected by the COVID-19 pandemic (Bechard et al., 2021). Brief online interventions can build resilience among healthcare workers (DeTore et al., 2021) and patients hospitalized due to COVID-19 (Shaygan et al., 2021). Strategies discussed above, such as stress appraisals, psychological flexibility, adaptive distractions and maintaining healthy relationships to mitigate burnout can be individualized to boost resilience.

### Suicide Prevention

Historical natural disasters such as pandemics and the literature examining their psychological impact have shown that although suicide rates may decline during the actual disaster period as communities come together to weather the storm, for the first and second year after life returns to normal, vulnerability to suicide may increase (Horney et al., 2020; Jafari et al., 2020). The COVID-19 pandemic has placed society in a unique position as far as historical dynamics have shown, with the sheer scale and specific day-to-day impact of similar pandemics not being experienced for just over a century (Morens et al., 2021). Specifically, the combination of required physical (and by extension social) isolation (Matthews et al., 2019) and the neuro-invasive potential of actual infection (Thye et al., 2022) might have created an optimal concoction of vulnerability to suicidal behaviour at a societal level. Beyond preventing direct deaths from infection, it is vital to explore preventing both the direct and indirect deaths related to the psychological impacts of COVID-19 through both a better understanding of the emerging evidence, and practical strategies.

#### Emerging Evidence

As rates of depression have increased over the course of the pandemic (Ettman et al., 2022), one of the potential risks that might accompany that rise would be suicidal behaviour and ideation. At first glance, suicidality is a concept that is deceptively simple to conceptualize in the context of COVID-19, but is actually composed of multiple distinct facets including suicidal ideation (thoughts), suicide attempts, and completed suicides (Dubé et al., 2021; Efstathiou et al., 2022; Moutier, 2021). An international study conducted by Pirkis et al. (2020) with over 20 countries examined suicide rates in the early months of the COVID-19 pandemic and found that there was no increase in suicide rates observed. Over a year later, this same observation was made by other studies despite the negative mental health impact that the COVID-19 pandemic and related health restrictions have had the world over (Appleby, 2021; Prati & Mancini, 2021).

More than two years into the pandemic, emerging evidence shows that depending on the country and nature of the study, there is variation in how suicidality has changed over time (Efstathiou et al., 2022). For instance, an international study by Schluter et al. (2022) examining patterns of suicidal ideation across eight countries in four continents found that suicidal ideation increased significantly over the course of the pandemic. Seemingly contradictory results in the literature could reflect how the rapidly changing nature of the pandemic has made it difficult to comprehensively keep up with internationally representative rates and scientifically rigorous assessments of all facets of suicidality (Banerjee et al., 2021; Smith et al., 2020). Indeed, Prati and Mancini (2021) caution that previous findings of a lack of increased suicide rates could reflect a false negative. This idea is supported by a later meta-analysis by Dubé et al. (2021) that found dramatically increased rates of self-harm, suicide ideation, and suicide attempts as the COVID-19 pandemic progressed, even compared to other meta-analyses that examined suicidality compared to pre-pandemic levels. This demonstrates the increased need for future research on COVID-19 related suicidal behaviour through multi-wave longitudinal, international, and rigorous culturally contextualized designs (Efstathiou et al., 2022).

#### Practical Applications

Given that the COVID-19 pandemic has persisted for over two years and emerging evidence showing increased rates of suicidality, it is imperative that preventative strategies for suicide be explored and incorporated. The tendency to focus on the worst feared outcomes of suicidality can overshadow findings that compared to before the pandemic, the demographics and reasons behind suicidality have shifted. Specifically, it has been found that women, male youth, single parents, the elderly, and those without previous psychopathology have been at growing risk for suicidality (Dubé et al., 2021; Kim et al., 2022; Rothman & Sher, 2021). This demonstrates that beyond the need for a multidisciplinary understanding of suicide trends at a broader level, interventions and strategies should be tailored to the demographics with the highest risk of suicidality (Holmes et al, 2020).

Wasserman et al. (2020) indicate that interventions can be introduced through education towards the awareness of suicidal behaviour for identifying help-seeking and reducing available means for suicide. Other methods of decreasing risk of suicidality include making crisis response systems more culturally contextualized (to be discussed later), strengthening social support on which individuals can rely during times of crisis, addressing COVID-19 related increases in drug and alcohol use, reducing access to deadly means, reducing financial stress, and limiting exposure to negative media (Efstathiou et al., 2022; Moutier, 2021). Additionally, people can develop protective factors by attending support groups and skill-building interventions (Wasserman et al., 2020). These precautions are particularly important and beneficial for vulnerable communities.

### Wellbeing of Marginalized Communities

Mental health does not exist in a vacuum but as an interaction between multiple risk-factors (Fiorillo & Gorwood, 2020; Mental Health Commission of Canada, 2009), with marginalized populations facing additional stressors such as discrimination and inequalities that contribute to pandemic-fatigue. For example, those with low-income, under-represented minorities, and first-generation students are more likely to face increased stressors at home (Liu et al., 2020). To blunt the community spread of variants, people need to be made aware that access to preventative measures, particularly for low wage essential workers, such as paid sick leave, decent facilities for self-quarantine, and vaccination priorities are win–win scenarios (Bowe et al., 2021; Busch et al, 2021; Shadmi et al., 2020).

With access to and competency using technology being the lifeline to the most up to date information, a means to stay connected with friends and loved ones, a way to continue attending school throughout the pandemic, and a valuable means of delivering tailored interventions – another barrier marginalized communities face is disproportionate access to technology. Beaunoyer et al. (2020) indicate that disparities in the quality, location of access, technical support, and access and expertise with technology can result in members of marginalized communities being unable to maintain a normal level of social contact because technologies that may have not been necessary pre-COVID-19 have become essential such as telehealth, remote learning, and even participating in critical life events such as prayers, funerals, weddings, and graduations virtually.

#### Emerging Evidence

Over the course of the COVID-19 pandemic, long present negative societal attitudes such as xenophobia have been further exposed (Abidin & Jing, 2020). Members of minority groups and those who have a low income are also more likely to have occupations that require working in-person, increasing their risk of exposure and sickness (Ali et al., 2020). Some studies have shown that forced proximity is a risk for aggression and domestic violence (Prime et al., 2020). These additional stressors contribute to pandemic-fatigue.

Accounting for these experiences requires practicing cultural humility, a process of self-reflection and discovery to build honest and trustworthy relationships (Yeager & Bauer-Wu, 2013). There are some existing technology-enabled mental health services and products that have been designed through a culturally humble lens to reduce barriers to care that marginalized communities face. These services and products include valuable features such as community-centered therapy and education, clinician cultural-humility training modules, and the ability to tailor interventions to different marginalized populations such as through modifying appropriate language (Schueller et al., 2019). However, these methods still require scaling, validity, and efficacy testing, and there are concerns on how these methods can be delivered to communities where access to required technology is limited (Akerele et al., 2021; Schueller et al., 2019).

#### Practical Applications

Behavioral health interventions should be customized for different racio-cultural groups (Novacek et al., 2020; van Bavel et al., 2020) and frontline healthcare workers (Greenberg et al., 2020) to account for the unique experiences they have faced throughout the COVID-19 pandemic (Pink et al., 2021). In line with emerging evidence, customized interventions for marginalized communities should be developed and applied using a cultural humility approach (Akerele et al., 2021). Previous research has placed particular emphasis on the idea that since those who are marginalized experience more stressful events in general, resilience interventions can be particularly effective at preventing psychopathology, including pandemic fatigue (Rashid et al., 2021; Spears, 2018). To that end, mindful attention to available community resources such as local support groups can be used to build social solidarity that can be particularly beneficial to disadvantaged populations (Mannarini et al., 2021).

It is critical to engage with marginalized communities to explore which specific policies and processes adversely impact their health and mental health. This can be reflected in practice by providing incentives, so members of any marginalized community feel valued and are provided with a platform to suggest concrete steps for establishing equitable health supports (Rashid & Di Genova, 2022). An example of this would be offering a variety of secure ways (e.g., text, flexible appointment times) to diverse communities in their languages so they can communicate effectively with health care professionals. Individuals from diverse gender and sexual identities and those who have been living in stigmatizing environments without family support, will benefit from non-traditional communication channels (Rashid & Di Genova, 2022). Finally, making a concerted effort to recruit, retain, develop, and promote staff from diverse backgrounds provides a systemic way of making the voices of marginalized community members heard. This diversity should go beyond tokenism: staff should have sophisticated, practical, and flexible skills to offer members of marginalized communities science-based factual information, but in culturally appropriate ways (Rashid & Di Genova, 2022).

## Conclusion

The COVID-19 pandemic — a watershed event of our lives — has lasted longer than we had hoped for or anticipated. In a matter of days, it changed our world and required us to be away from each other to keep a virus at bay. For more than two years, social distancing, mask mandates, working and studying remotely, vacillating between variants and vaccines, most of us experienced stress, signs, and symptoms of pandemic fatigue.

In this paper we unpack how specific resilience and wellbeing strategies can help us to manage pandemic fatigue. Gleaned from published and emerging research, we share specific strategies such as learning to reappraise stressors and exercise cognitive flexibility, strengthening our relationships in proximal settings, and more equitable distribution of opportunities and resources for our marginalized communities impacted disproportionately by the COVID-19 pandemic. This is our cautious understanding and interpretation of the literature as the rapidly evolving nature of the pandemic has placed restrictions on the ability to conduct rigorous methodology and limited the emergence of sophisticated designs to further clarify the construct of pandemic fatigue. With time, sophisticated designs to further clarify and operationalize a nuanced and fine-grained construct of pandemic fatigue will shed light on the causes and consequences. Future studies and meta-analyses should differentiate between general population level examinations and the impact on specific sub-groups within those populations which may be experiencing a differential impact.

Acknowledging the pain, grief, and uncertainty that may ensue for years to come, the COVID-19 pandemic could reflect our collective efforts to either buckle down under misinformation and pessimism, or systemically cultivate, maintain, and refine resilience and wellbeing. Only our individual resolve, shared identities, and responsibilities towards our planet and its inhabitants can turn these ideas into pragmatic actions.

## Appendix 1. Pandemic fatigue strategies tables

Table 1

**Table 1:** Pandemic fatigue strategies table

Author(s)	Article Title	Strategy
Fear from Personal Risk of Infection		
Tannenbaum et al., 2015	Appealing to fear: A meta-analysis of fear appeal effectiveness and theories	Strong or intense fear produces the greatest behaviour change only when people feel a sense of efficacy and vice-versa, with stronger self-efficacy reducing fear
Hou et al., 2020a, b	Self-efficacy and fatigue among non-frontline health care workers during COVID-19 outbreak: A moderated mediation model of posttraumatic stress disorder symptoms and negative coping	An important balancing act needs to be done internally regarding Covid-19 related fears to prevent the scenarios of people either feeling that the risk to their own and loved one's health are overblown (too little fear) or that their COVID-19 related fears are so present and invasive that their ability to successfully perform in these burdensome circumstances are severely impaired (too much fear)
Negative Visualization & Stress Appraisals		
White et al., 2019	Focusing on the future from afar: Self-distancing from future stressors facilitates adaptive coping	When we visualize potential negative outcomes related to COVID-19, we should mentally distance ourselves from what is happening now from what may happen in the future
Robertson & Codd, 2019	Stoic philosophy as a cognitive-behavioral therapy	View potential negative Covid-19 related outcomes in terms of logical visualizations instead of emotional ones. Also attempt to reduce the vividness of our imaginations because that allows us to view potential future events in a more constructive manner
Psychological Flexibility		
McCracken et al., 2021	The role of psychological flexibility in the context of COVID-19: Associations with depression, anxiety, and insomnia	To increase psychological flexibility, currently existing brief training methods such as ACT self-help could be used
Optimizing Intimate Relationships		
Prime et al., 2020	Risk and resilience in family well-being during the COVID-19 pandemic	To reduce the potential burden of extended time in proximity, relationships in the family should be forged and maintained to offset the consequences of otherwise distressing situations such as loneliness. Also, collaboration within the family should take place to reach a shared framework of how COVID-19 related stressors impact each family member to increase compassion and understanding between household members
Promoting Early Childhood Independence		
Swit & Breen, 2022	Parenting During a Pandemic: Predictors of Parental Burnout	Interventions to promote early childhood independence may provide an avenue to protect parents from parental burnout by helping parents balance increased responsibilities that came with the pandemic. Parents can also themselves try and encourage independence in their children from a younger age
Enriched Online Connections		
Rashid & McGrath, 2020	Strengths-based actions to enhance wellbeing in the time of COVID-19	Individuals can be advised to share digital literature (e.g., e-books) and content with people they know to make them happy, providing both a reason and topic to speak on
Nowland et al., 2018	Loneliness and social internet use: pathways to reconnection in a digital world?	Healthy internet use can have a positive impact on feelings of loneliness when it is used to form new relationships or improve existing ones instead of avoiding socialization
Notable Virtual Interventions		
Williams et al., 2021	Interventions to reduce social isolation and loneliness during COVID-19 physical distancing measures: A rapid systematic review	There are many potentially low-cost and digitally deliverable interventions are effective, including; mindfulness-based therapies, visual art discussions, laughter therapy, and Tai Chi Qigong meditation Variation in educational programme interventions demonstrated the positive effects of addressing social integration barriers and making friends had on loneliness which can be targeted directly in a therapeutic context
Healthy Use of Virtual Communication Platforms		
Baker & Murphy, 2021	Conducting Successful Virtual Meetings While Managing COVID Fatigue	To avoid negative outcomes of video communication, look at the camera, instead of other participants to establish direct eye contact and to create an authoritative response; keeping your background simple and clean, signalizing professionalism; staying mute when not speaking to avoid unfortunate interruptions and use slightly louder than normal voice when speaking, as if you are presenting to a larger audience; avoiding side activities, to stay fully engaged in the meeting; and asking to keep the video feed off while not speaking in order to eliminate distraction and overstimulation
Social Media		
Shao et al., 2021	Social Media and Emotional Burnout Regulation During the COVID-19 Pandemic: Multilevel Approach	To harness social media for positive coping, people should be educated on effective ways to improve emotional regulation and try to consume more emotionally positive or neutral content
Resilience		
Crum et al., 2013; Crum & Lyddy, 2014	Rethinking stress: The role of mindsets in determining the stress response (2013) De-stressing stress: The power of mindsets and the art of stressing mindfully (2014)	People should identify how their stressful experiences can be used to increase psychological toughness. This can take the form of helping people to reorganize priorities, identify what they can do and cannot do, and harness the stress response to focus on positive gains
Mindfulness Practice		
Crum et al., 2013; Crum & Lyddy, 2014	Rethinking stress: The role of mindsets in determining the stress response (2013) De-stressing stress: The power of mindsets and the art of stressing mindfully (2014)	When maladaptive coping mechanisms stem from an unhealthy mindset, viewing one’s stressors through a lens of mindfulness can allow that stress to be enhancing instead of debilitating. In the case of COVID-related anticipatory grief, this may mean acknowledging that the COVID-19 pandemic is causing concern for your loved ones, recognizing that it is normal to feel elevated stress related to the worry for loved one’s health during a pandemic, and finally identifying how the circumstances surrounding the stressors can be used as motivation to avoid the outcomes that are feared
Adaptive Distractions		
Janson & Rohleder, 2017	Distraction coping predicts better cortisol recovery after acute psychosocial stress	Individuals can distract themselves by focusing on things which they can control. Teaching people healthy coping strategies can help in mitigate the impact of negative symptomatic stress. Distraction and seeking emotional support have been shown as effective coping strategies in young adults such as students when they have limitations on their behaviour (e.g., physical distancing, staying home)
Reducing Burnout		
Hou et al., 2020a, b	Self-efficacy and fatigue among non-frontline health care workers during COVID-19 outbreak: A moderated mediation model of posttraumatic stress disorder symptoms and negative coping	Limiting extended work hours, improving sleep quality, and increasing the use of social supports has been found to be related to improved self-efficacy during the Covid-19 pandemic
Reflection & Planning		
Oettingen et al., 2015	Self‐regulation of time management: Mental contrasting with implementation intentions	One can reflect on an activity they find meaningful and that can realistically be done under the current circumstances. Then you can formulate a clear plan regarding how you would start doing the activity tomorrow. Consider the necessary steps to ensure that you are ready to start pursuing the activity and to think about any obstacles that could stop you from doing this activity and how to overcome them, etc
Journaling & Meditation		
Jackson Preston, 2022	We must practice what we preach: a framework to promote well-being and sustainable performance in the public health workforce in the United States	Reflection through journaling and mediation can be powerful tools to address burnout by making it easier to identify stressors that can then addressed
Individualized Wellness Plans		
Stamm, 2005 Butler & McClain-Meeder, 2015	The ProQOL manual (the professional quality of life scale) Self-Care Starter Kit	Developing an individualized wellness plan that integrates various burnout prevention strategies can make integration into daily life easier. Various free and easy to access tools exist that are designed to help people understand and develop their own personal wellness plan, such as the Professional Quality of Life (proQOL) Measure and University of Buffalo self-care starter kit
Psychoeducation		
Hansel et al., 2020	Behavioral Health and Response for COVID-19	Psychoeducation can be used to identify maladaptive coping strategies such as substance abuse and provide healthier alternative strategies. These strategies may be reflected in messaging on financial coping, providing information on how to recognize symptoms of prolonged grief and trauma in oneself and others, and emphasizing the role of community support in coping while social distancing
Keeping Interventions Dynamic		
Novacek et al., 2020	Mental health ramifications of the COVID-19 pandemic for Black Americans: Clinical and research recommendations	The implementation and content of behavioral health interventions should be customized for different racio-cultural groups
Community Support and Resources		
Mannarini et al., 2021	The potential of psychological connectedness: Mitigating the impacts of COVID-19 through sense of community and community resilience	Mindful attention to available community resources such as local support groups can be used to build social solidarity that can be particularly beneficial to disadvantaged populations
Understanding the Needs of Marginalized Communities		
Rashid & Di Genova, 2022	Campus Mental Health Across Canada in 2020–21: The Ongoing Impact of COVID-19. Perspectives from student affairs leaders	Providing individuals from diverse gender and sexual identities, marginalized communities, and those who have been living in stigmatizing environments without family support, benefit from the ability to explain their needs through non-traditional communication channels An additional concerted effort to recruit, retain, develop, and promote staff from diverse backgrounds provides a systemic way of making the voices of marginalized community members heard. This diversity should go beyond tokenism: staff should have sophisticated, practical, and flexible skills to offer members of marginalized communities science based factual information, but in culturally appropriate ways
Suicidality Recognition		
Wasserman et al., 2020	Adaptation of evidence‐based suicide prevention strategies during and after the COVID‐19 pandemic	Interventions can be introduced through education towards the awareness of suicide to identify help-seeking behaviours and reduce available means for suicide. Additionally, people can develop protective factors by attending support groups and skill-building interventions
Suicidality Prevention		
Efstathiou et al., 2022 Moutier, 2021	Suicidality and COVID-19: Suicidal ideation, suicidal behaviors and completed suicides amidst the COVID-19 pandemic (Review) Suicide Prevention in the COVID-19 Era: Transforming Threat Into Opportunity	Methods of decreasing risk of suicidality include making crisis response systems more culturally contextualized, strengthening social support on which individuals can rely during times of crisis, addressing COVID-19 related increases in drug and alcohol use, reducing access to deadly means, reducing financial stress, and limiting exposure to negative media
